# Pheophorbide A and Paclitaxel Bioresponsive Nanoparticles as Double-Punch Platform for Cancer Therapy

**DOI:** 10.3390/pharmaceutics13081130

**Published:** 2021-07-23

**Authors:** Francesca Moret, Luca Menilli, Manuele Battan, Daniele Tedesco, Marta Columbaro, Andrea Guerrini, Greta Avancini, Claudia Ferroni, Greta Varchi

**Affiliations:** 1 Department of Biology, University of Padova, 35100 Padova, Italy; francesca.moret@unipd.it (F.M.); luca.menilli@unipd.it (L.M.); greta.avancini@phd.unipd.it (G.A.); 2 Institute for the Organic Synthesis and Photoreactivity—Italian National Research Council, 40121 Bologna, Italy; manuele.battan@gmail.com (M.B.); daniele.tedesco@isof.cnr.it (D.T.); andrea.guerrini@isof.cnr.it (A.G.); 3 IRCCS Istituto Ortopedico Rizzoli, 40121 Bologna, Italy; marta.columbaro@ior.it

**Keywords:** prodrug, paclitaxel, pheophorbide A, nanoparticles, tumor microenvironment, photodynamic therapy

## Abstract

Cancer therapy is still a challenging issue. To address this, the combination of anticancer drugs with other therapeutic modalities, such as light-triggered therapies, has emerged as a promising approach, primarily when both active ingredients are provided within a single nanosystem. Herein, we describe the unprecedented preparation of tumor microenvironment (TME) responsive nanoparticles exclusively composed of a paclitaxel (PTX) prodrug and the photosensitizer pheophorbide A (PheoA), e.g., PheoA≅PTX_2_S. This system aimed to achieve both the TME-triggered and controlled release of PTX and the synergistic/additive effect by PheoA-mediated photodynamic therapy. PheoA≅PTX_2_S were produced in a simple one-pot process, exhibiting excellent reproducibility, stability, and the ability to load up to 100% PTX and 40% of PheoA. Exposure of PheoA≅PTX_2_S nanoparticles to TME-mimicked environment provided fast disassembly compared to normal conditions, leading to PTX and PheoA release and consequently elevated cytotoxicity. Our data indicate that PheoA incorporation into nanoparticles prevents its aggregation, thus providing a greater extent of ROS and singlet oxygen production. Importantly, in SK-OV-3 cells, PheoA≅PTX_2_S allowed a 30-fold PTX dose reduction and a 3-fold dose reduction of PheoA. Our data confirm that prodrug-based nanocarriers represent valuable and sustainable drug delivery systems, possibly reducing toxicity and expediting preclinical and clinical translation.

## 1. Introduction

Cancer is a highly complex disease that develops through a multistep carcinogenesis process involving several cellular pathways, and despite the numerous advances in treatment options, cancer therapy is still challenging [1]. Combination therapy, consisting of the administration of two or more anti-cancer drugs, represents an essential milestone in tumor therapy, having the ability to tackle multi drug resistance (MDR), and enhance the treatment efficacy by targeting key pathways in a synergistic or at least additive manner [2]. However, the use of anticancer agents, alone or in combination, faces critical drawbacks related to their inadequate spatiotemporal release kinetics and non-selective cytotoxicity, which determines the insurgence of life-threatening side effects [3].

In this view, the combination of anticancer drugs with other therapeutic modalities, such as light-triggered therapies, has emerged as a promising and less invasive approach resulting in significant cancer cells eradication [4]. Light is a mighty mean for the local and non-invasive activation of therapeutic agents at the desired site [5,6], through a controlled and timely dosage of the released species, without affecting physiological parameters such as temperature, pH, and ionic strength. Among light-activated treatment modalities, photodynamic therapy (PDT) has been approved for the treatment of certain tumors or is in the advanced stage of clinical trials [7,8]. In PDT, cancer cells are destroyed by reactive oxygen species (ROS), formed upon the irradiation of a photo-active molecules, e.g., photosensitizers (PSs), at a specific wavelength and in presence of oxygen [9,10]. To enable a better therapeutic outcome, concepts of combining PDT and chemotherapy have been developed by means of nanotechnological approaches [11]. The combination of PDT and chemotherapy has gained increasing attention thanks to its potential to induce antitumor immunity [12] and to revert MDR caused by the chemotherapeutic agent [11].

Recently, we demonstrated that combining chemotherapeutic drugs, such as paclitaxel (PTX) [13], docetaxel [14] and salinomycin [10] with the PS chlorin e6 co-loaded into keratin nanoparticles, synergistically enhances the treatment efficacy on different solid tumors, e.g., osteosarcoma, cervix epithelioid carcinoma and breast cancer, both in 2D and 3D in vitro models. In addition, studies from other authors indicate that the co-delivery of a PS and an anticancer drug exhibits improved cytotoxicity and less adverse side effects in animal models as compared to their free form [15,16,17].

Despite the promising outcomes in preclinical investigations of nanocarriers-based drug delivery systems, their translation to the clinic has been limited, primarily because of the reduced benefit to patients despite the highly specific and expensive technologies involved [18]. On the other hand, drug derivatization techniques are increasingly used to develop prodrugs and modular platforms aimed at reducing differences among the physicochemical properties of bioactive molecules, improving their release predictability, and reducing systemic exposure and side effects [19].

The tumor microenvironment (TME) is characterized by mildly acidic conditions, hypoxia, and strongly altered levels of glutathione (GSH) and hydrogen peroxide (H_2_O_2_), making these characteristics exploitable for a tumor-selective drug release [20]. In particular, GSH levels in bloodstream are found to be in the micromolar range (2–20 μM), while its concentration is shown to be up to 3 orders of magnitude higher (2–10 mM) in the TME, thus representing a unique condition for designing selective tumor-releasing entities. In this context, the use of bioresponsive prodrugs of first-line chemotherapeutics could meaningfully improve drug pharmacokinetics and solubility, while limiting systemic exposure and side effects [21,22].

PTX, an antimitotic chemotherapeutic agent, has been widely used for the treatment of breast, ovarian, non-small cell lung cancers and Kaposi’s sarcoma; however, its application has been limited due to its poor water solubility and severe systemic toxicity [23,24,25]. PTX tends to crystallize in aqueous solution, owing to π−π interactions of aromatic rings [26]; to overcome this restraint, the insertion of a flexible linker between two PTX molecules has shown to decrease the drug’s crystallinity. The freely rotatable σ chain is supposed to increase intermolecular hydrophobic interactions, making the PTX structure less rigid and preventing the formation of drug aggregates/crystals. Besides reducing PTX molecules packaging, the linker could also promote core cross-linking and facilitate its self-assembly into nanoparticles [27]. Dimeric prodrugs could improve the stability of nano-formulations, acting as crossing agents and increasing loading efficiency [28]. Recently, Pei et al. described a PTX dimer with a thioether linker (PTX_2_S), which is able to self-assemble into uniform nanovesicles with a very impressive PTX loading (94%), a 2000-fold enhancement in solubility compared to free PTX, and an excellent GSH responsiveness [29]. This redox sensitive dimer has shown to overcome several drawbacks associated with conventional PTX delivery nanoformulations, such as the risk of premature leakage in bloodstream, the low effective drug amount, and the high tendency to crystallize [30].

In this work, we describe the unprecedented preparation of nanoparticles exclusively composed of a PTX prodrug and the PS pheophorbide A (PheoA), with the aim of achieving both a TME-triggered, controlled release of PTX by means of a redox-sensitive thioether linkage, and a synergistic/additive effect with PheoA-mediated PDT. Our prodrug-assembled nanoparticles (PheoA≅PTX_2_S) can be produced in a simple, one-pot process and exhibit the impressive ability to load up to 100% of PTX and 40% of PheoA, leading to a dual approach for anticancer therapy. The absence of additional exogeneous carriers to achieve our formulation constitutes an essential feature for its faster translation to pre-clinical and clinical application. PheoA≅PTX_2_S nanoparticles were characterized in terms of dimensions, morphology, physiological stability, ROS, and singlet oxygen production. Furthermore, we investigated in vitro the anticancer activity of PheoA≅PTX_2_S by studying drug release and cytotoxicity in simulated TME conditions and the combination therapy potential in breast and ovarian cancer cell models.

## 2. Materials and Methods

### 2.1. Materials

All reagents were used as obtained from commercial sources unless otherwise indicated. Solvents were dried over standard drying agents and freshly distilled prior to use. Ultrapure water was produced using a Sartorius Arium Pro^®^ system (Sartorius, Monza, Italy). ^1^H NMR spectra were recorded on 500 MHz Varian spectrometer and deuterated chloroform was used as the solvent. ^1^H chemical shifts values (δ) are referenced to the residual nondeuterated components of the NMR solvents (δ = 7.26 ppm for CHCl_3_). Flash chromatography was performed on Teledyne Isco CombiFlash Rf 200 using RediSep normal-phase silica flash columns (230–400 mesh). TLC was performed on silica gel 60 F254 plastic sheets. Pure PTX was purchased from TCI Europe. All compounds tested in biological assays were >95% pure, as determined by HPLC–UV analysis (Waters 600 HPLC instrument connected to photodiode array detector 996). The purity of intermediates was >90%, unless otherwise stated. Absorption spectra were recorded using a Cary 100 UV–Vis spectrophotometer (Agilent Technologies, Milan, Italy).

### 2.2. Synthesis of PTX_2_S

The PTX dimer bridged with a thioether linker (abbreviated as PTX_2_S) was synthesized as previously reported [29]. Briefly, PTX (99 mg, 0.12 mmol, 1 equation.) was dissolved in anhydrous DCM (2.0 mL) in a three-necked flask, then 2,2′-thiodiacetic acid (13 mg, 0.085 mmol, 0.73 eq), EDC⋅HCl (46 mg, 0.24 mmol, 2 eq) and DMAP (1.5 mg, 0.012 mmol, 0.1 eq) were added under an argon atmosphere. After stirring for 1 h at room temperature, an additional 23 mg of EDC⋅HCl (0.12 mmol, 1 eq) and 1.5 mg di DMAP (0.012 mmol, 0.1 eq) were added. The resulting mixture was stirred at room temperature for an additional 4 h. The solvent was removed under pressure and the crude material was purified on a silica gel column eluted with 30% ethyl acetate in DCM, affording 95 mg of a white solid, yield = 88% (see supporting information for NMR spectrum, Figure S1).

^1^H NMR (500 MHz, CDCl_3_) δ 8.16 (d, *J* = 7.4 Hz, 4H), 7.74 (d, *J* = 7.6 Hz, 4H), 7.66–7.58 (m, 2H), 7.56–7.49 (m, 4 H), 7.48–7.31 (m, 16 H), 7.26–7.21 (m, *NH*, 2 H), 6.34–6.19 (m, 4 H), 6.09 (dd, *J* = 9.3, 2.5 Hz, 2 H), 5.68 (d, *J* = 7.1 Hz, 2 H), 5.53 (d, *J* = 2.9 Hz, 2 H), 4.98 (d, *J* = 8.6 Hz, 2 H), 4.43 (dd, *J* = 10.8, 6.7 Hz, 2 H), 4.32 (d, *J* = 8.4 Hz, 2 H), 4.21 (d, *J* = 8.4 Hz, 2 H), 3.81 (d, *J* = 7.0 Hz, 2 H), 3.27 (d, *J* = 14.7. Hz, 2 H), 3.16 (d, *J* = 14.7. Hz, 2 H), 2.63–2.52 (m, 2 H), 2.49 (s, 6 H), 2.39 (dd, *J* = 15.3, 9.4 Hz, 2 H), 2.22 (s, 6 H), 2.19–2.12 (m, 2 H), 1.89 (s, 6 H), 1.69 (s, 6 H), 1.22 (s, 6 H), 1.14 (s, 6 H).

### 2.3. Preparation of PTX_2_S Nanoparticles (mPTX_2_S)

mPTX_2_S were prepared by a modified nanoprecipitation procedure [31]. Briefly, 50 μL of a PTX_2_S solution in DMSO (10 mg/mL) were slowly injected into 0.8 mL of water, under vigorous stirring. After 10 min, the solution was analyzed through DLS (400 μL of mPTX_2_S in 1.6 mL of water). The nanoparticles suspension was stored at 0 °C.

### 2.4. Preparation of PTX_2_S Nanoparticles Loaded with PheoA (PheoA≅PTX_2_S)

PheoA≅PTX_2_S were prepared through the previously mentioned nanoprecipitation method. One hundred μL of a PheoA solution in DMSO (1.5 mg/mL) were added to 50 μL of a PTX_2_S solution in DMSO (10 mg/mL). This mixture was slowly injected into 1.9 mL of water, under vigorous stirring. After 10 min, the solution was analyzed through DLS and stored in the dark at 0 °C and used for the following in vitro stability studies (Section 2.6). DMSO removal was performed by dialysis (MWCO 12–14 kDa) or ultrafiltration (Amicon^®^ Ultra filters, MWCO 100 kDa).

### 2.5. Characterization of Nanoparticles

The hydrodynamic diameter and polydispersity index (PDI) of nanoparticles in aqueous solution (0.5 mg/mL) were determined by dynamic light scattering (DLS) analysis at 25 °C using a NanoBrook Omni Particle Size Analyzer (Brookhaven Instruments Corporation, New York, NY, USA) equipped with a 35-mW red diode laser (nominal wavelength 640 nm). Electrophoretic mobility, i.e., ζ-potential, was measured at 25 °C using the same instrument. The morphology of nanoparticles was analyzed by transmission electron microscopy (TEM): nanoparticles (0.1 mg/mL) were dispensed as a drop on a carbon-coated nickel grid and after 20 min, any excess of the solution was absorbed by filter paper. The nanoformulation was subsequently observed with a Jeol Jem-1011 transmission electron microscope (Jeol Jem, Peabody, MA, USA).

### 2.6. In Vitro Stability of Nanoparticles

In vitro stability studies were performed over time (5 days) at 37 °C (i) in phosphate buffered saline (PBS) solution, (ii) in PBS solution containing 10% recombinant human serum albumin (HSA), and (iii) in PBS solution containing 20% fetal bovine serum (FBS). The changes in particle size distribution were monitored by DLS.

### 2.7. Reactive Oxygen Species and Singlet Oxygen Generation

The ROS production was evaluated for free PheoA and PheoA≅PTX_2_S using the chemical probe 2′,7′-dichlorodihydrofluorescein diacetate (H_2_DCFDA) [32]. Indeed, in the presence of ROS, the nonfluorescent molecule H_2_DCFDA is first hydrolyzed to 2,7-dichlorodihydrofluorescein (H_2_DCF) and then oxidized to the highly fluorescent species DCF. In details, H_2_DCFDA was dissolved in methanol obtaining a 1.1 mM solution. Two mL of NaOH (0.01 M) were then added to 500 μL of this solution and stirred for 30 min at room temperature; afterwards, 10 mL of phosphate buffer (pH = 7.4) were added providing the ROS probe solution. Samples were prepared as follows: 127 μL PheoA≅PTX_2_S in water (0.25 mg/mL) were added (PheoA final concentration: 12.4 μM) to a cuvette containing 155 μL of water, 500 μL of phosphate buffer, and 218 μL of ROS probe, as previously prepared. For measuring ROS production in the presence of albumin, 135 μL of HSA dissolved in water (35 mg/mL) were added to 0.5 mL of a PheoA≅PTX_2_S aqueous solution (0.25 mg/mL). The mixture was incubated at 37 °C for 1 h in the dark, then, 161 μL were withdrawn and then added to a cuvette containing 121 μL of water, 500 μL of phosphate buffer, and 218 μL of ROS probe. Both solutions were irradiated with a Tungsten lamp (Phillips, 300 W) at a distance of 40 cm up to 30 min and the absorption spectra were recorded at each time point with a Cary 100 UV–Vis spectrophotometer (Agilent Technologies), reading the increase in absorbance at 500 nm. Singlet oxygen generation was determined by using the chemical probe 9,10-dimethylanthracene (DMA) [33]. Quartz cells (0.75 mL) with a 1 cm path length and containing 50 μL of PheoA≅PTX_2_S (PheoA = 4 μg/mL) and 650 μL of DMA in dimethylformamide (35 μM) were irradiated with a tungsten lamp (Phillips, 300 W) at 20 cm for different irradiation times. Absorption spectra of the solution were recorded every minute for 10 min reading the decrease in absorbance at 378 nm.

### 2.8. GSH or H_2_O_2_-Triggered Nanoparticles Disassembly

The in vitro nanoparticles disassembly was evaluated by treating 1.2 mL PheoA≅PTX_2_S (0.25 mg/mL) with or without GSH (10 μM or 10 mM), with or without H_2_O_2_ (300 μM and 500 μM), irradiated or not with a tungsten lamp (Phillips, 300 W) at a distance of 40 cm. The changes in particle size distribution were monitored by DLS analysis.

### 2.9. Cell Lines

MDA-MB-231 (human triple negative breast cancer), SK-OV-3 (human ovarian carcinoma) and CCD-34Lu (human normal lung fibroblasts) were purchased from American Type Culture Collection (ATCC, Rockville, MA, USA). MDA-MB-231 and CCD-34Lu cells were grown in DMEM with Glutamax^TM^ supplemented with 10% heat inactivated FBS, 100 U/mL streptomycin, and 100 µg/mL penicillin G while SK-OV-3 cells were grown in RPMI ATCC formulated medium supplemented with 10% FBS and antibiotics. Cells were maintained at 37 °C under a humidified atmosphere, containing 5% CO_2_. Cell culture medium and supplements were purchased from Life Technologies (Italy), while sterile plasticwares were from Falcon^®^ (Corning, Glendale, AZ, USA).

### 2.10. Cytotoxicity of Free PTX and mPTX_2_S toward Cancer and Normal Cells

The cytotoxicity of PTX, either delivered as free drug (dissolved in DMSO) or as mPTX_2_S was assessed with the MTS assay (CellTiter 96^®^ AQueous One Solution Cell Proliferation Assay, Promega, Milan, Italy) in cancer (MDA-MB-231, SK-OV-3) and normal (CCD-34Lu) cells exposed to increasing drug concentrations. Cells (8 × 10^3^ cells/well for MDA-MB-231, 7 × 10^3^ cells/well SKOV-3, 6 × 10^3^ cells/well for CCD-34Lu) were seeded in 96-well plates, and after 24 h the medium was replaced with a fresh one containing the drug delivered in free form or entrapped in nanoparticles. To evaluate PTX cytotoxicity, cell viability was measured after 24 h of cell incubation with the drug formulations and an additional 24 h of cell release in drug-free medium (incubation time 24 h + 24 h). For MTS assay, the medium was replaced with 100 μL of serum-free medium and 20 μL of the CellTiter 96^®^ reagent. After 1 h, the absorbance at 492 nm was measured with a Multiskan Go (Thermo Fischer Scientific, Carlsbad, CA, USA) plate reader and the cell viability was expressed as a function of absorbance relative to that of control cells (considered as 100% viability).

### 2.11. In-Cell Simulation of Redox Environment (Experiment with GSH-OEt)

In order to recapitulate the reducing environment typical of TME by further increasing the GSH level already present in the cancer cells medium, experiments were performed in which the medium was supplemented with glutathione reduced ethyl ester (GSH-OEt, Sigma Aldrich, Saint Louis, MO, USA) [34]. Cells were seeded as described in 2.11 and pre-treated with 10 mM GSH-OEt for 90 min. At the end of the pre-incubation time, cells were treated with nanoparticles for 5 h and released in drug-free medium for a further 24 h before being assessed for viability with MTS assay. If foreseen, after the 5 h of incubation with nanoparticles, cells were irradiated in PBS with red light (600–750 nm) emitted by a PDT1200 lamp (Waldmann, Villingen-Schwenningen, Germany), and with a total fluence of 1 J/cm^2^ at a power density of 20 mW/cm^2^.

### 2.12. Combination Therapy Experiments

For combination therapy experiments, cells were seeded as described in paragraph 2.11 and treated with increasing concentrations of PTX, PheoA or their combination (PTX/PheoA ratio 2.2:1, *w*/*w*), delivered as free drugs or loaded into nanoparticles. Cell viability was measured with MTS assay 24 h after drug incubation and an additional 24 h in which the cells were kept in drug-free medium (dark cytotoxicity; time point 24 h + 24 h). For photo-toxicity experiments (PDT in vitro), cells were seeded and treated as described above, and at the end of the 24 h period, cells were washed twice with PBS Ca^2+^ and Mg^2+^ and irradiated with red light (600–750 nm) emitted by a PDT1200 lamp with a total fluence of 1 J/cm^2^. The power density was 20 mW/cm^2^ as measured with the radiometer PDT 1200 L (Waldmann). After irradiation and upon replacement of PBS with fresh medium, cells were incubated in the dark for 24 h prior the assessment of cells viability by MTS assay (phototoxicity; time point 24 h + 24 h). Moreover, in order to determine if the combined treatment, e.g., PTX-chemotherapy and PheoA-PDT, resulted in a synergistic effect, CI values were calculated using the CompuSyn software (ComboSyn Inc., New Jersey, NJ, USA) based on the Chou and Talalay method [35]. From the experimental data on cell viability, the Fraction affected (Fa) values were derived for each drug concentration and the data were processed by the CompuSyn software as described in detail in [36]. For each drug and drugs combination, the software also calculated the drug concentration that inhibits cell survival by 50% (IC_50_ value).

### 2.13. Cellular Uptake and Localization of PheoA and PheoA≅PTX_2_S

The internalization rate of PheoA loaded in nanoparticles or delivered to cells as free drug was measured by flow cytometry while the intracellular localization was studied by confocal microscopy. For uptake studies, cells (5 × 10^4^ cells/well for SKOV-3 and 6 × 10^4^ cells/well for MDA-MB-231) were grown in 24-well plates for 24 h and incubated for 1 or 4 h with 0.1 or 0.25 μM PheoA as free drug or loaded in PheoA≅PTX_2_S. At the end of the incubation time, cells were washed twice with Versene, detached from the plates with trypsin that was neutralized by the addition of FBS. Cells were centrifuged and resuspended in Versene before measuring PheoA fluorescence using a BD Fortessa^TM^ X-20 flow cytometer (Becton Dickinson, San Jose, CA, USA). A red laser (640 nm) was used to excite the PS and its fluorescence was detected at wavelengths >670 nm (APC channel). For each sample, 10^4^ events were acquired and analyzed using the FACSDiva and FlowJo softwares.

For intracellular localization studies, 6 × 10^4^ MDA-MB-231 cells were grown in 35 mm imaging dishes (Eppendorf AG, Hamburg, Germany) for 24 h and incubated for 4 h with 1 μM of PheoA or PheoA≅PTX_2_S. Fifteen minutes before completing the incubation, cells were stained MitoTracker^®^ Green FM (0.1 μM), or ER-Tracker^TM^ Green (1 μM), used as a marker for mitochondria and endoplasmic reticulum, respectively. Cells were then washed twice with HBSS and observed with a Leica SP5 confocal microscope; acquired images were analyzed using ImageJ software.

### 2.14. Annexin/PI Assay

Cells (6 × 10^4^) were seeded in 24-well plates; after 24 h, cells were treated with drugs delivered as standard formulations or included in nanoparticles. At the end of the incubation time (24 h), and 12 h after cell irradiation (1 J/cm^2^), cells were detached from the plates with trypsin, collected in flow cytometry tubes, washed with PBS, and centrifuged. Annexin V, previously diluted in binding buffer, was added to each tube, and the cells were incubated in the dark for 10 min at room temperature, washed with the binding buffer, and then propidium iodide (PI) (20 μg/mL) was added before performing flow cytometry analysis.

## 3. Results and Discussion

### 3.1. Synthesis and Characterization of mPTX_2_S and PheoA≅PTX_2_S

**Figure 1:**
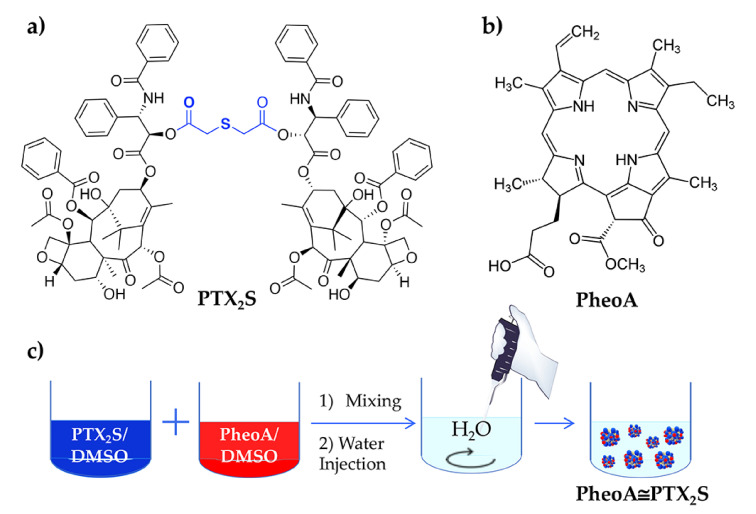
(**a**) Chemical structure of PTX_2_S; (**b**) Chemical structure of PheoA; (**c**) Schematic representation of PheoA≅PTX_2_S preparation by the nanoprecipitation method.

In this study, we report the unprecedented preparation of bioresponsive nanoparticles assembled from a PTX prodrug, e.g., PTX_2_S (Figure 1a) and loaded with a high amount of the hydrophobic PS PheoA (Figure 1b), with the aim of enhancing drugs solubility in water, improving the selective accumulation and release of PTX at the tumor tissue, and assessing the potential of their combination in different cancer cell lines. Indeed, bimodal strategy based on chemotherapeutic agents in association with PDT is recognized to increase the outcome of cancer therapy, especially when drugs are co-delivered into a single nanoformulation [36,37]. Therefore, we synthesize a dimeric prodrug of PTX (PTX_2_S) bearing a thioether linker that could be specifically cleaved at the TME thanks to the presence of elevated GSH and ROS concentrations [31]. In our approach, the intrinsic specificity of PDT is combined to the TME-responsivity of PTX_2_S-based nanoparticles that allows PTX release and delivery preferentially at the tumor site. Of note, biocompatible and high tissue-penetrating red light is used as an activation source for PheoA and as a further stimulus to increase PTX release upon ROS production.

PTX_2_S was obtained according to a one-step literature procedure [29], while nanoparticles were produced through a modified nanoprecipitation method, consisting in the slow addition of a DMSO solution of PTX_2_S (10 mg/mL) into water under vigorous stirring. The formed mPTX_2_S exhibited an average hydrodynamic diameter of about 80 nm, a ζ-potential of –29 mV and a narrow dispersity of 0.085.

**Figure 2:**
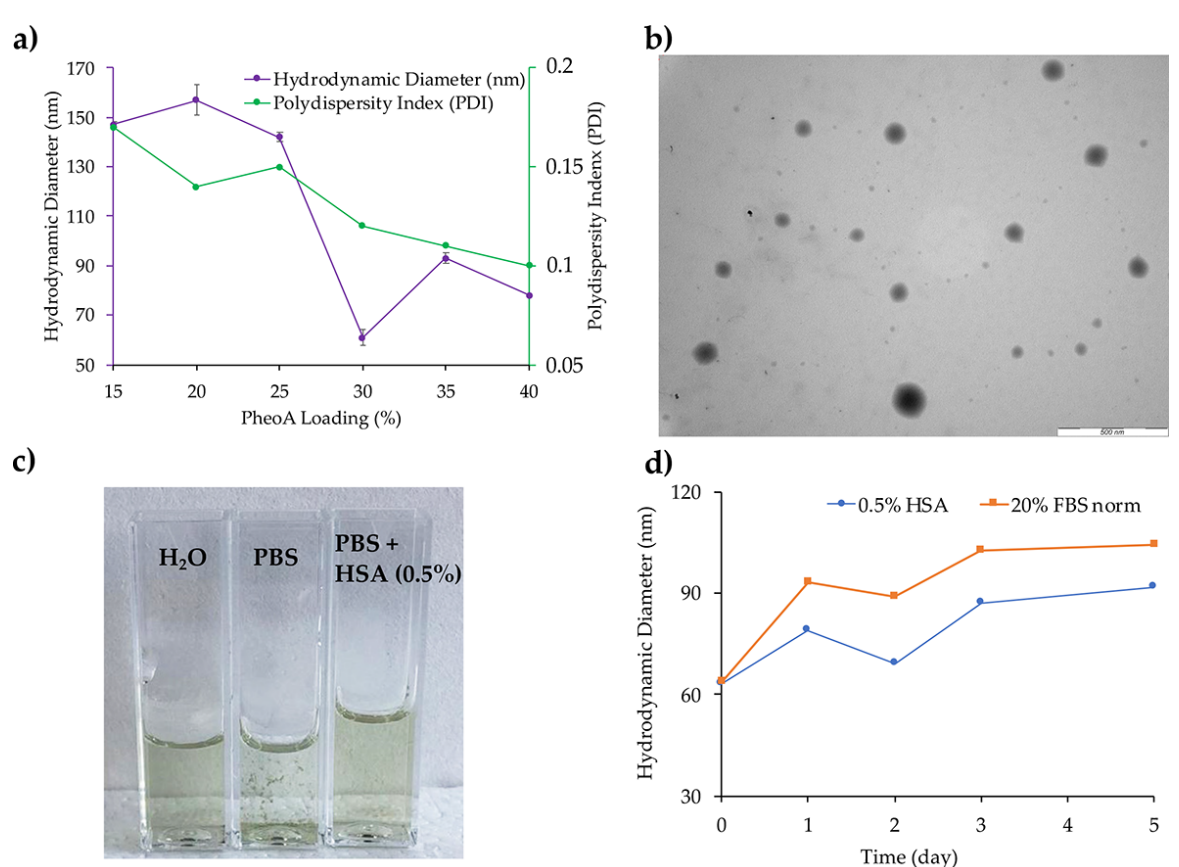
(**a**) PheoA≅PTX_2_S optimization as a function of PheoA loading; (**b**) TEM analysis of PheoA≅PTX_2_S (scale bar 500 nm; (**c**) Comparison between PheoA≅PTX_2_S solution in water, PBS and PBS + 0.5% HSA; (**d**) PheoA≅PTX_2_S stability trend in PBS + 0.5% HSA and PBS + 20% FBS.

To load the PS into nanoparticles, different amounts of PheoA dissolved in DMSO (1.5 mg/mL) were mixed to a DMSO solution of PTX_2_S (10 mg/mL), and subsequently injected in milliQ water (Figure 1c). An optimization study allowed to fine-tune both PheoA loading and nanoparticles size, indicating that a PheoA loading up to 40% (W_PheoA_/W_PTX2S_) was very well tolerated (Table S1). However, an optimal PheoA≅PTX_2_S preparation was achieved with a 30% PheoA content, which was in turn preferred for the following characterization and in vitro experiments. Interestingly, our results showed that, by increasing PheoA loading, nanoparticles size considerably decreased, while the polydispersity index (PDI) did not change to a great extent (Figure 2a), very likely because the hydrophobic nature of the PS contributed to further nanoparticles’ stabilization. Under these conditions, stable and reproducible PheoA≅PTX_2_S nanoparticles were obtained with average hydrodynamic diameter of 61 nm, ζ-potential of −30 mV and PDI of 0.12, indicating the presence of a single and highly monodisperse nanoparticles population.

PheoA≅PTX_2_S morphology was further investigated through transmission electron microscopy (TEM), which confirmed their regular and spherical shape (Figure 2b). The average dry diameter detected by TEM was of 60 nm, in good agreement with DLS results. Furthermore, removal of DMSO by dialysis or ultrafiltration did not affect nanoparticles hydrodynamic diameters, confirming their excellent storage stability in aqueous solution for over 1 month (data not shown).

### 3.2. Stability of mPTX_2_S and PheoA≅PTX_2_S

Stability studies were performed by monitoring nanoparticles size at 37 °C. Remarkably, in PBS solution at pH 7.4, PheoA≅PTX_2_S immediately precipitated (Figure 2c), while the presence of 0.5% HSA or 20% FBS in the buffer conferred an extraordinary colloidal stability as indicated in Figure 2c,d. This behavior might indicate the instant formation of a stabilizing protein corona around the nanoparticles, which could be of particular relevance for a future in vivo application.

As shown in Figure 2d, in the presence of 0.5% HSA (blue line) a slight size increase from 62 to 92 nm occurred over 5 days, while no significant changes were observed in the polydispersity index. As expected, in the presence of 20% FBS, the hydrodynamic diameter increased more significantly, e.g., from 64 to 104 nm, over the same observation time (Figure 2d, orange line), most likely due to the interaction between nanoparticles and serum proteins. It is worth noting that the presence of serum proteins does not induce the formation of aggregates, as confirmed by the constant PDI (0.2–0.3); on the contrary, it considerably improves nanoparticles’ stability as compared to saline solution. These data are in good agreement with literature studies on protein corona formation on other types of nanoparticles, [37] and indicate that PheoA≅PTX_2_S nanoparticles have good affinity for serum proteins, allowing their colloidal stabilization. In principle, these preliminary data suggest that our formulations might be optimized for in vivo application by pre-coating nanoparticles with HSA to properly control hard protein corona formation, ultimately allowing to increase their blood circulation time and tumor cells internalization [38].

### 3.3. ROS and ^1^O_2_ Generation

As PheoA≅PTX_2_S is conceived as a chemotherapeutic and PDT nanoplatform, the efficiency of ROS generation after light irradiation was assessed by measuring the increase in the absorption peak at 500 nm of the probe 2,7-dichlorofluorescein (DCF) [32]. Solutions containing PheoA≅PTX_2_S, with or without HSA, and H_2_DCF (see materials and methods) were irradiated with a Tungsten lamp at a distance of 40 cm and absorption spectra measured at different irradiation time intervals.

**Figure 3:**
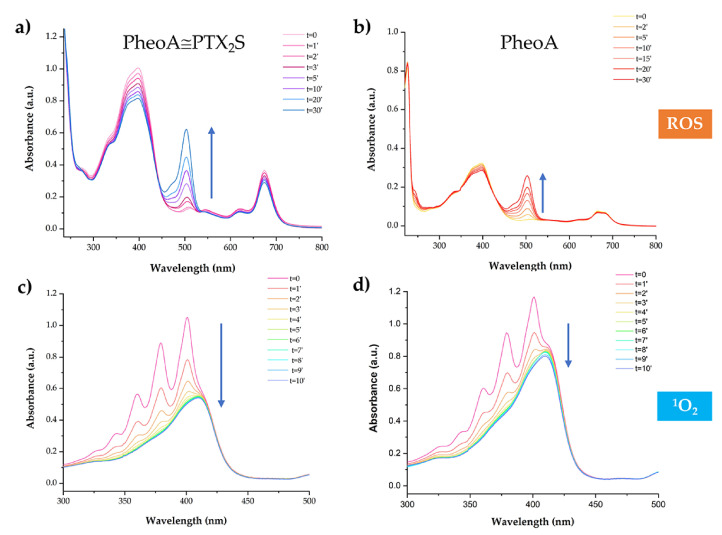
PheoA≅PTX_2_S ability to produce ROS and ^1^O_2_ upon light irradiation. Absorption spectra of 2′,7′-dichlorofluorescein (DCF) measured at different irradiation times in the presence of (**a**) PheoA≅PTX_2_S and (**b**) free PheoA. ^1^O_2_ analysis performed at different irradiation times of a solution of (**c**) DMA + PheoA≅PTX_2_S and (**d**) DMA + PheoA.

The increase of the absorption band at 500 nm indicated that ROS formation was light and dose-dependent (Figure 3a). Remarkably, an equal amount of PheoA, e.g., 12.4 μM, loaded onto nanoparticles produced two-fold more ROS as compared to PheoA as free form (Figure 3b). In presence of HSA, ROS production slightly decreased due to the antioxidant properties of serum albumin [39]; however, as reported in the literature and confirmed by in vitro experiments, ROS generation induced by PDT was not influenced by the protein corona (data not shown) [40,41]. ^1^O_2_ generation efficiency was evaluated by monitoring the decrease of the absorption peak at 378 nm of the DMA probe upon irradiation; in the presence of ^1^O_2_, DMA is converted to its nonfluorescent endoperoxide form, thus resulting in absorbance decrease [42]. As shown in Figure 3c, DMA absorbance steadily decreased by increasing the irradiation time up to 10 min, confirming the capability of PheoA≅PTX_2_S to produce ^1^O_2_ with high efficiency. As for ROS production, our results confirm that once loaded into nanoparticles, PheoA’s ability to induce ^1^O_2_ upon light irradiation is considerably higher with respect to its free form (Figure 3d), most likely due to PS protection from self-quenching and aggregation phenomena.

### 3.4. GSH and H_2_O_2_ Triggered Disassembly of PheoA≅PTX_2_S Nanoparticles

We next investigated nanoparticles disassembly in the presence of GSH and H_2_O_2_ as reductive and oxidative triggers, respectively. In details, nanoparticles were incubated at 37 °C with two different GSH concentrations: 10 mM for mimicking natural TME and 10 μM as reference value for normal tissue. As expected, at 10 mM GSH, PheoA≅PTX_2_S nanoparticles disassembled already after 2 h from incubation, leading to a white precipitate formation. On the contrary, when incubated with 10 μM GSH, nanovesicles resulted to be stable over 24 h, as confirmed by DLS measurement (Figure S2a). These data are in complete agreement with previously reported data on the same PTX dimer [26,29]. Besides the GSH response, we also monitored nanoparticles’ disassembly under oxidative conditions: the size of PheoA≅PTX_2_S showed a remarkable increase in the presence of 300 μM H_2_O_2_ after 36 h, while 500 μM H_2_O_2_ treatment gave similar size change already after 15 h (Figure S2b) with precipitate formation, indicating that high H_2_O_2_ concentrations promoted the disruption processes of the nanoparticles. Nanoparticles’ disassembly further increased when treated with 500 μM H_2_O_2_ and irradiated with a Tungsten lamp at a distance of 40 cm for 20′, thus indicating that concomitant PDT action could improve drug release and overall efficacy (Figure S2b).

### 3.5. Cytotoxicity of Nanoparticles in Cancer and Normal Cells In Vitro

To dissect the capability of PTX to be selectively released from mPTX_2_S in the reductive TME, we compared the cytotoxicity exerted by PTX and mPTX_2_S in two cancer cell lines of different origin, i.e., breast adenocarcinoma MDA-MB-231 and ovarian carcinoma SK-OV-3, and on a non-cancerous cell line, i.e., normal lung fibroblasts CCD-34-Lu.

**Figure 4:**
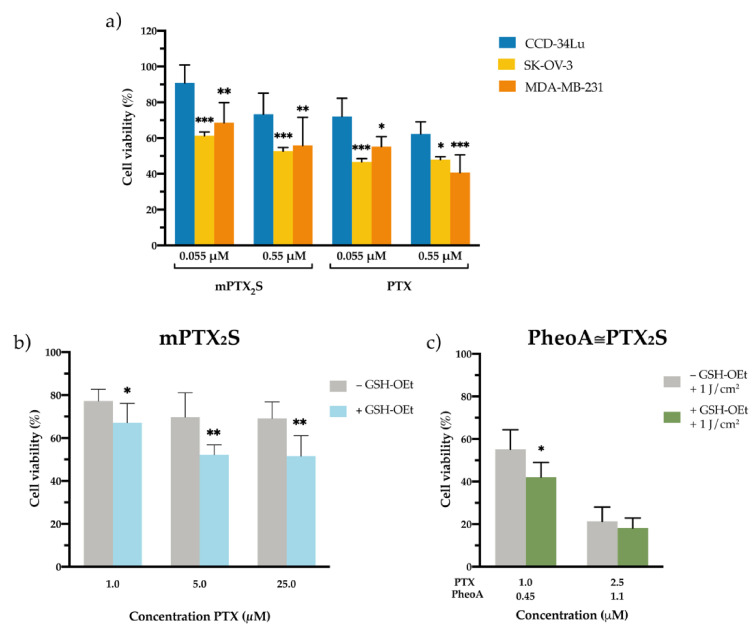
In vitro cytotoxicity of nanoparticles in different microenvironments. (**a**) Cell viability measured in normal fibroblasts (CCD-34-Lu) and cancer cells (breast MDA-MB-231 and ovarian SK-OV-3) incubated for 24 h with increasing concentrations of mPTX_2_S or PTX delivered in the standard solvent and measured 24 h post cell-release in drug-free medium. * *p* < 0.05; ** *p* < 0.01; *** *p* < 0.001 significantly different from CCD-34Lu (ANOVA One-way with Bonferroni’s correction). (**b**) Cytotoxicity induced by mPTX_2_S measured in MDA-MB-231 cells pre-treated or not with GSH-OEt (90 min) before nanoparticles incubation (5 h), to further increase the reductive microenvironment in vitro. (**c**) Cytotoxicity measured in MDA-MB-231 cells (pre-treated or not with GSH-OEt) incubated with PheoA≅PTX_2_S and irradiated with red light (1 J/cm^2^). * *p* < 0.05; ** *p* < 0.01 significantly different from-GSH-OEt (Student’s *t*-test). All data in the figure are expressed as mean ± S.D. of at least two independent experiments carried out in triplicate.

Cells were treated for 24 h with mPTX_2_S and released for an additional 24 h in nanoparticles-free medium before assessing viability with MTS assay. As expected, cell viability was reduced to higher extent in both cancer cell lines, while the viability of fibroblasts was only scarcely affected by mPTX_2_S treatment (Figure 4a). Of note, when cells were incubated with PTX delivered in standard solvent (DMSO), cytotoxicity was slightly increased in all the three cell lines (Figure 4a). These results strongly support our hypothesis that micellar PTX_2_S prodrug is potentially less toxic and more selective compared to free PTX since it requires more time and appropriate conditions to release the pharmacologically active drug. Moreover, to further tread in vitro the simulation of the in vivo TME [34,43], MDA-MB-231 cancer cells were pre-incubated with 10 mM GSH-OEt, followed by a 5 h treatment with mPTX_2_S (Figure 4b). As a result, cell viability was significantly reduced when the culture medium was supplemented with GSH-OEt, confirming that a more reductive microenvironment promotes a greater extent of PTX release and consequently cell death.

Besides the reductive potential of TME in promoting drug release, we tested whether the combination of GSH-OEt with the ROS produced during PDT treatment, could further enhance the cytotoxic effect of PheoA≅PTX_2_S. Therefore, MDA-MB-231 cells, pre-treated or not with GSH-OEt, were incubated with PheoA≅PTX_2_S for 5 h and subsequently irradiated with red light at a total dose of 1 J/cm^2^. As reported in Figure 4c, light irradiation combined with GSH pre-treatment significantly increased the extent of cell death, at least at the lowest concentration tested, suggesting a higher degree of PTX release under these conditions, as already appreciated during release experiments (Figure S2).

### 3.6. In Vitro Combination Therapy with PheoA≅PTX_2_S Nanoparticles

Based on the previous results, which account for a satisfactory bio-responsivity of PheoA≅PTX_2_S nanoparticles both under redox conditions and upon ROS-production, we next investigated the anticancer potential of combining PTX antimitotic activity with PheoA-based PDT. To this end, MDA-MB-231 and SK-OV-3 cells were incubated for 24 h with PheoA≅PTX_2_S (PTX/PheoA ratio 2.2:1, *w*/*w*, corresponding to a 30% PheoA loading), or as a combination of the free drugs in the same ratio, and compared with single drug/prodrug treatment (mPTX_2_S, PheoA, PTX). Cells were either maintained in the dark or irradiated with red light (total dose 1 J/cm^2^) and assessed for cell viability 24 h post-irradiation. Importantly, dark cytotoxicity is ascribable exclusively to PTX action, since PheoA was not able to reduce cell viability to any extent (Figure S3). Cell viability curves of all tested PTX formulations showed a similar trend, including a sharp viability decrease at low PTX doses, which reaches a plateau around the IC_50_ values (Figure S3).

**Figure 5:**
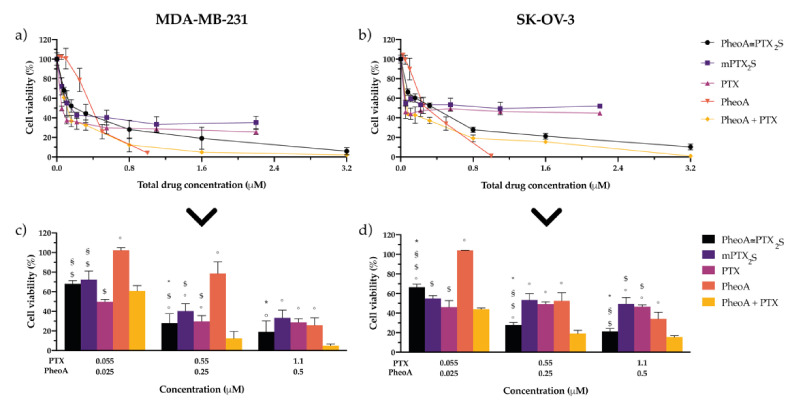
In vitro combination therapy. Dose-response curves of (**a**) MDA-MB-231 or (**b**) SK-OV-3 cells incubated for 24 h with single drugs or their combination delivered free or in nanoparticles and irradiated with 1 J/cm^2^ of light; after an additional 24 h in drug-free medium, cell viability was measured by MTS assay. Total drug concentration is referred to PTX + PheoA concentration. Data are expressed as mean percentage ±SD of at least three independent experiments, carried out in triplicate. To better compare the cytotoxic effects of the different drug formulations, some concentrations have been extrapolated: panel (**c**) for MDA-MB-231, and panel (**d**) for SK-OV-3 cells. Statistical significance was calculated applying the ANOVA Two-way with Bonferroni’s correction: * significantly different from mPTX_2_S; ^§^ significantly different from PTX; ^$^ significantly different from PheoA; ° significantly different from PheoA + PTX.

Upon light irradiation, the cytotoxic effect determined by PTX was potentiated by PheoA-induced cell mortality (Figure 5a,b). The PDT effect exerted by PheoA, either as a free drug or combined with PTX, induced a complete cell killing in both cell lines. To better appreciate the contribution elicited by each single drug or drugs combination, specific drugs concentrations from Figure 5a,b were extrapolated and reported in Figure 5c,d. PTX delivered as standard formulation displayed the highest cytotoxicity in both cell lines (IC_50_ of 0.028 μM in MDA-MB-231 and 0.053 μM in SK-OV-3, Table 1). As expected, mPTX_2_S exerted a lower cytotoxic effect that well correlates with the specific environmental conditions and prolonged times required to promote PTX release at the tumor site, thus ultimately confirming our initial hypothesis. Accordingly, when combination therapy was performed, the IC_50_ values of PheoA≅PTX_2_S were two-fold higher as compared to PTX + PheoA (Table 1) in both cell lines.

**Figure 6:**
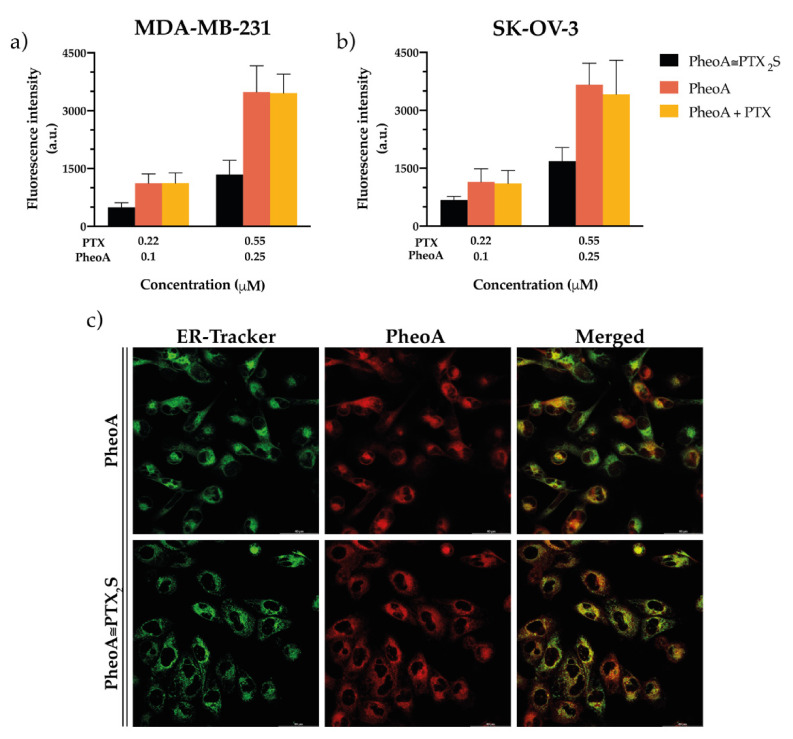
In vitro uptake and intracellular localization studies. Flow cytometry measurements of the intracellular uptake of PheoA delivered in the different formulations in (**a**) MDA-MB-231 and (**b**) SK-OV-3 cells exposed to the treatments for 4 h. Data are expressed as mean percentage ± SD of at least three independent experiments, carried out in triplicate. (**c**) Confocal microscopy images of MDA-MB-231 cells showing the co-localization between the red fluorescence of PheoA (delivered in the standard solvent or loaded in PheoA≅PTX_2_S) and the green fluorescence of ER-Tracker used as a specific probe for endoplasmic reticulum. Scale bars: 40 µm.

The percentage of cell death induced by the free drugs combination (Figure 5c,d, yellow bar) is only slightly superior to that elicited by PheoA≅PTX_2_S (Figure 5c,d, black bar). Interestingly, internalization studies performed by flow cytometry both on cells treated with free drugs and PheoA≅PTX_2_S (Figure 6a,b), indicate that free PheoA, alone or combined with PTX, was internalized twice compared to PheoA delivered in nanoparticles. These results indicate that, despite the higher internalization extent, PheoA alone undergoes aggregation and stacking phenomena, which in turn reduce its phototoxic activity. Conversely, notwithstanding the halved intracellular content, PheoA loaded into nanoparticles preserves its phototoxic potential and ability to induce a similar degree of cell mortality. These in vitro findings are in good agreement with cell-free ROS and singlet oxygen production results (Figure 3), showing that PheoA≅PTX_2_S can produce more significant amounts of oxygen radicals compared to the same concentrations of free PheoA, which undergoes aggregation in aqueous media (Figure 3b).

Confocal microscopy studies performed on MDA-MB-231 cells revealed that, regardless of the PheoA formulation (i.e., standard solvent or PheoA≅PTX_2_S), the PS is mainly localized in the endoplasmic reticulum (Figure 6c), thus indicating that the oxidative stress generated after PDT treatment targeted the same intracellular sites, irrespective of PheoA delivery. Of note, differently from other literature reports [44,45], we were not able to observe an exclusive mitochondrial localization for PheoA (Figure S4), very likely due to the differences in treatment protocols and cell lines used.

Nevertheless, being the site of ROS production unaffected by PheoA delivery modality and being PTX prodrug less efficient in inducing cytotoxicity with respect to standard PTX, it could be speculated that PheoA loaded into nanoparticles retains a higher phototoxic activity, most likely due to lower or no aggregation phenomena.

In order to assess the type of interaction (i.e., antagonistic, additive, or synergic combination) that occurs when PTX and PheoA-PDT are combined, the Compusyn analysis was performed based on the cytotoxicity data reported in Figure 5a,b. The Compusyn software is based on the Chou and Talalay method, which analyzes drugs interaction and allows to calculate the Combination Index (CI) using the median-effect principle [35], where CI < 1 indicates synergism; CI ≈ 1, additivity; and CI > 1, antagonism. The occurrence of eventual drugs synergism for our combination (free PTX + free PheoA or PheoA@PTX_2_S) was determined by plotting the CI vs. the fraction of affected cells (Fa) (i.e., killed cells). In particular, for PheoA≅PTX_2_S, the results in the Fa-CI plots (Figure S5a,b) were obtained by comparing the Fa values with respect to the following combinations: (i) free PTX +f ree PheoA; (ii) mPTX_2_S +. free PheoA. Our results indicate that the combination of free PTX and free PheoA was synergic for Fa higher than 0.5 (Figure S4, blue line). At the same time, when loaded into nanoparticles, drugs’ interaction was primarily synergic in SK-OV-3 cells, irrespectively if compared to free PTX +free PheoA (red curve) or mPTX_2_S + free PheoA (green curve). On the other hand, in MDA-MB-231 cells, treatment with PheoA≅PTX_2_S was synergic exclusively for Fa comprised between 0.7 and 0.9. Interestingly, the analysis of the Dose Reduction Index (DRI) values (Table 1) for Fa equal to 0.5, reveals that in MDA-MB-231 cells, both the combinations of free drugs and the use of PheoA≅PTX_2_S exclusively allows for a PheoA dose reduction (PheoA DRI = 9.06; PheoA + PTX or PheoA≅PTX_2_S DRI = 5.30). Notably, the combination therapy with PheoA≅PTX_2_S in SK-OV-3 cells allowed a 30 times PTX dose reduction (PTX DRI = 31.6) with respect to the use of mPTX_2_S and free PheoA, and a three times PheoA dose reduction (PheoA DRI = 3.19).

**Figure 7:**
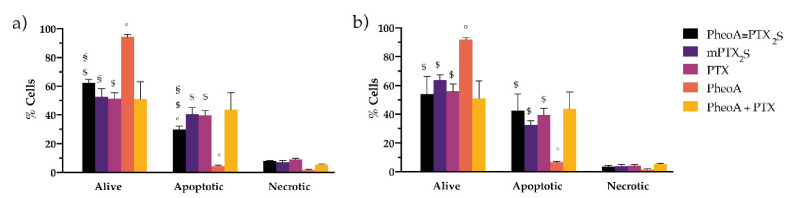
In vitro studies on cell death mechanism. MDA-MB-231 (**a**) and SK-OV-3 (**b**) cells incubated with the different drug formulations for 24 h, irradiated with 1 J/cm^2^ of red light and stained with the Annexin V/PI kit 12 h post-irradiation. Data are expressed as mean percentage ±SD of at least two independent experiments, carried out in triplicate. Statistical significance was calculated applying the ANOVA Two-way with Bonferroni’s correction: ^§^ significantly different from PTX; ^$^ significantly different from PheoA; ° significantly different from PheoA + PTX.

Preliminary experiments on cell death mechanism elicited by the different drug formulations/combinations revealed that, in cells treated for 24 h, exposed to light and stained with the Annexin V/PI kit 12 h post-irradiation: (i) the prevailing mechanisms for PTX-induced cell death is apoptosis; (ii) the effects of PheoA-PDT are not appreciable at least at this observation time and for the selected drug dose; (iii) the extent of apoptosis induced by the combination of the free drugs and by PheoA≅PTX_2_S is quite comparable (Figure 7), thus excluding different cell death mechanisms based on the different drugs delivery modality.

## 4. Conclusions

We have herein reported the straightforward in-water preparation of nanoparticles exclusively composed of the PTX_2_S prodrug and loaded with a high content of PheoA (30%). Nanoparticles showed excellent reproducibility and stability, especially in the presence of serum proteins. Importantly, when PheoA≅PTX_2_S are exposed to TME-mimicked GSH or ROS concentrations, both in cell-free and in cell cultures in vitro, they promptly disassemble, leading to PTX (and PheoA) release, in turn resulting in elevated cytotoxicity.

Moreover, our data indicate that PheoA incorporation into nanoparticles most likely prevents the photosensitizer’s aggregation, thus providing a higher extent of ROS and singlet oxygen production. Indeed, despite the limited synergic effect observed in both cell lines, the use of PheoA≅PTX_2_S in SK-OV-3 allows for a 30-fold dose reduction of PTX and a 3-fold dose reduction of PheoA, thus in principle reducing the overall systemic toxicity, but unaffecting the tumor efficacy. Remarkably, the presence of HSA or FBS dramatically improved PheoA≅PTX_2_S colloidal stability, suggesting an extraordinary affinity of our nanoparticles for serum proteins. These data collectively confirm that prodrug-based nanocarriers represent valuable and sustainable systems for drug formulation and delivery, avoiding the use of additional exogenous materials and stabilizers, thus expediting their translation into preclinical and clinical validation.

Although the present study produced significant achievements and indications about our nanosystem, future in vivo preclinical studies have been planned to establish whether this nanoformulation is suitable for further clinical application. Indeed, due to the complexity of the human organism and the co-existence of several physiological barriers, the selective accumulation of nanocarriers within the tumor might require incorporating an additional targeting element. In this view, the present work already prompts us to consider the preparation of nanoparticles to exploit endogenous serum albumin as a selective and biomimetic carrier.

## Figures and Tables

**Table 1:** IC_50_ and Dose-Reduction Index (DRI) values calculated by the Compusyn analysis of cytotoxicity data (Figure 5a,b) of MDA-MB-231 and SK-OV-3 cells exposed to the different drug formulations/combinations. The DRI values were calculated for cells exposed to combination therapy and indicate how many folds the concentration of each single drug can be reduced to obtain a survival value (Fa, Fraction affected) of 50%.

Drug Formulation	IC_50_ (μM)		DRI			
	MDA-MB-231	SK-OV-3	MDA-MB-231		SK-OV-3	
			PheoA	PTX	PheoA	PTX
mPTX_2_S	0.15	5.72	-	-	-	-
PTX	0.028	0.053	-	-	-	-
PheoA	0.35	0.26	-	-	-	-
PheoA + PTX	0.12	0.12	9.06	0.32	6.88	0.63
PheoA≅PTX_2_S	0.21	0.26	5.30	1.02	3.19	31.6

## Data Availability

Not applicable.

## Supplementary Materials

The following data are available online at https://www.mdpi.com/article/10.3390/pharmaceutics13081130/s1, Table S1: Nanoparticles’ optimization studies, Figure S1: PTX2S 1HNMR spectrum, Figure S2: Nanoparticles’ disassembly under different conditions, Figure S3: In vitro dark cytotoxicity, Figure S4: In vitro intracellular localization studies, Figure S5: Combination index analysis.

## References

[1] Misra R., Acharya S., Sahoo S.K. (2010). Cancer nanotechnology: Application of nanotechnology in cancer therapy. Drug Discov. Today.

[2] Bayat Mokhtari R., Homayouni T.S., Baluch N., Morgatskaya E., Kumar S., Das B., Yeger H. (2017). Combination therapy in combating cancer. Oncotarget.

[3] Zuluaga M.-F., Lange N. (2008). Combination of Photodynamic Therapy with Anti-Cancer Agents. Curr. Med. Chem..

[4] Cao J., Chen Z., Chi J., Sun Y., Sun Y. (2018). Recent progress in synergistic chemotherapy and phototherapy by targeted drug delivery systems for cancer treatment. Artif. Cells Nanomed. Biotechnol..

[5] Brown S.B., Brown E.A., Walker I. (2004). The present and future role of photodynamic therapy in cancer treatment. Lancet Oncol..

[6] Celli J.P., Spring B.Q., Rizvi I., Evans C.L., Samkoe K.S., Verma S., Pogue B.W., Hasan T. (2010). Imaging and Photodynamic Therapy: Mechanisms, Monitoring, and Optimization. Chem. Rev..

[7] Dos Santos A.F., De Almeida D.R.Q., Terra L.F., Baptista M.S., Labriola L. (2019). Photodynamic therapy in cancer treatment—An update review. J. Cancer Metastasis Treat..

[8] Zhang J., Jiang C., Figueiró Longo J.P., Azevedo R.B., Zhang H., Muehlmann L.A. (2018). An updated overview on the development of new photosensitizers for anticancer photodynamic therapy. Acta Pharm. Sin. B.

[9] Ferroni C., Del Rio A., Martini C., Manoni E., Varchi G. (2019). Light-Induced Therapies for Prostate Cancer Treatment. Front. Chem..

[10] Avancini G., Guerrini A., Ferroni C., Tedesco D., Ballestri M., Columbaro M., Menilli L., Reddi E., Costa R., Leanza L. (2021). Keratin nanoparticles and photodynamic therapy enhance the anticancer stem cells activity of salinomycin. Mater. Sci. Eng. C.

[11] Khdair A., Chen D., Patil Y., Ma L., Dou Q.P., Shekhar M.P.V., Panyam J. (2010). Nanoparticle-mediated combination chemotherapy and photodynamic therapy overcomes tumor drug resistance. J. Control. Release.

[12] Castano A.P., Mroz P., Wu M.X., Hamblin M.R. (2008). Photodynamic therapy plus low-dose cyclophosphamide generates antitumor immunity in a mouse model. Proc. Natl. Acad. Sci. USA.

[13] Martella E., Ferroni C., Guerrini A., Ballestri M., Columbaro M., Santi S., Sotgiu G., Serra M., Donati D.M., Lucarelli E. (2018). Functionalized Keratin as Nanotechnology-Based Drug Delivery System for the Pharmacological Treatment of Osteosarcoma. Int. J. Mol. Sci..

[14] Gaio E., Guerrini A., Ballestri M., Varchi G., Ferroni C., Martella E., Columbaro M., Moret F., Reddi E. (2019). Keratin nanoparticles co-delivering Docetaxel and Chlorin e6 promote synergic interaction between chemo- and photo-dynamic therapies. J. Photochem. Photobiol. B..

[15] Chang J.E., Yoon I.S., Sun P.L., Yi E., Jheon S., Shim C.K. (2014). Anticancer efficacy of photodynamic therapy with hematoporphyrin-modified, doxorubicin-loaded nanoparticles in liver cancer. J. Photochem. Photobiol. B Biol..

[16] Luo D., Carter K.A., Miranda D., Lovell J.F. (2017). Chemophototherapy: An Emerging Treatment Option for Solid Tumors. Adv. Sci..

[17] Pedrosa P., Mendes R., Cabral R., Martins L.M.D.R.S., Baptista P.V., Fernandes A.R. (2018). Combination of chemotherapy and Au-nanoparticle photothermy in the visible light to tackle doxorubicin resistance in cancer cells. Sci. Rep..

[18] Maeda H., Khatami M. (2018). Analyses of repeated failures in cancer therapy for solid tumors: Poor tumor-selective drug delivery, low therapeutic efficacy and unsustainable costs. Clin. Transl. Med..

[19] van der Meel R., Sulheim E., Shi Y., Kiessling F., Mulder W.J.M., Lammers T. (2019). Smart cancer nanomedicine. Nat. Nanotechnol..

[20] Kennedy L., Sandhu J.K., Harper M.-E., Cuperlovic-Culf M. (2020). Role of glutathione in cancer: From mechanisms to therapies. Biomolecules.

[21] He X., Cai K., Zhang Y., Lu Y., Guo Q., Zhang Y., Liu L., Ruan C., Chen Q., Chen X. (2018). Dimeric Prodrug Self-Delivery Nanoparticles with Enhanced Drug Loading and Bioreduction Responsiveness for Targeted Cancer Therapy. ACS Appl. Mater. Interfaces.

[22] Li S., Shan X., Wang Y., Chen Q., Sun J., He Z., Sun B., Luo C. (2020). Dimeric prodrug-based nanomedicines for cancer therapy. J. Control. Release.

[23] Singla A.K., Garg A., Aggarwal D. (2002). Paclitaxel and its formulations. Int. J. Pharm..

[24] Gornstein E., Schwarz T.L. (2014). The paradox of paclitaxel neurotoxicity: Mechanisms and unanswered questions. Neuropharmacology.

[25] Gelderblom H., Verweij J., Nooter K., Sparreboom A. (2001). Cremophor EL: The drawbacks and advantages of vehicle selection for drug formulation. Eur. J. Cancer.

[26] Pei Q., Hu X., Zheng X., Xia R., Liu S., Xie Z., Jing X. (2019). Albumin-bound paclitaxel dimeric prodrug nanoparticles with tumor redox heterogeneity-triggered drug release for synergistic photothermal/chemotherapy. Nano Res..

[27] Meng Z., Lv Q., Lu J., Yao H., Lv X., Jiang F., Lu A., Zhang G. (2016). Prodrug Strategies for Paclitaxel. Int. J. Mol. Sci..

[28] Han X., Chen J., Jiang M., Zhang N., Na K., Luo C., Zhang R., Sun M., Lin G., Zhang R. (2016). Paclitaxel-Paclitaxel Prodrug Nanoassembly as a Versatile Nanoplatform for Combinational Cancer Therapy. ACS Appl. Mater. Interfaces.

[29] Pei Q., Hu X., Zhou J., Liu S., Xie Z. (2017). Glutathione-responsive paclitaxel dimer nanovesicles with high drug content. Biomater. Sci..

[30] Luo C., Sun J., Liu D., Sun B., Miao L., Musetti S., Li J., Han X., Du Y., Li L. (2016). Self-Assembled Redox Dual-Responsive Prodrug-Nanosystem Formed by Single Thioether-Bridged Paclitaxel-Fatty Acid Conjugate for Cancer Chemotherapy. Nano Lett..

[31] Wang J., Pei Q., Xia R., Liu S., Hu X., Xie Z., Jing X. (2020). Comparison of Redox Responsiveness and Antitumor Capability of Paclitaxel Dimeric Nanoparticles with Different Linkers. Chem. Mater..

[32] Ferroni C., Sotgiu G., Sagnella A., Varchi G., Guerrini A., Giuri D., Polo E., Orlandi V.T., Marras E., Gariboldi M. (2016). Wool Keratin 3D Scaffolds with Light-Triggered Antimicrobial Activity. Biomacromolecules.

[33] Ballestri M., Caruso E., Guerrini A., Ferroni C., Banfi S., Gariboldi M., Monti E., Sotgiu G., Varchi G. (2018). Core–shell poly-methyl methacrylate nanoparticles covalently functionalized with a non-symmetric porphyrin for anticancer photodynamic therapy. J. Photochem. Photobiol. B Biol..

[34] Yuan L., Chen W., Hu J., Zhang J.Z., Yang D. (2013). Mechanistic Study of the Covalent Loading of Paclitaxel via Disulfide Linkers for Controlled Drug Release. Langmuir.

[35] Chou T.-C. (2006). Theoretical basis, experimental design, and computerized simulation of synergism and antagonism in drug combination studies. Pharmacol. Rev..

[36] Gaio E., Conte C., Esposito D., Miotto G., Quaglia F., Moret F., Reddi E. (2018). Co-delivery of Docetaxel and Disulfonate Tetraphenyl Chlorin in One Nanoparticle Produces Strong Synergism between Chemo- and Photodynamic Therapy in Drug-Sensitive and -Resistant Cancer Cells. Mol. Pharm..

[37] Partikel K., Korte R., Mulac D., Humpf H.-U., Langer K. (2019). Serum type and concentration both affect the protein-corona composition of PLGA nanoparticles. Beilstein J. Nanotechnol..

[38] Rampado R., Crotti S., Caliceti P., Pucciarelli S., Agostini M. (2020). Recent Advances in Understanding the Protein Corona of Nanoparticles and in the Formulation of “Stealthy” Nanomaterials. Front. Bioeng. Biotechnol..

[39] Roche M., Rondeau P., Singh N.R., Tarnus E., Bourdon E. (2008). The antioxidant properties of serum albumin. FEBS Lett..

[40] Yeo E.L.L., Cheah J.U.J., Thong P.S.P., Soo K.C., Kah J.C.Y. (2019). Gold Nanorods Coated with Apolipoprotein E Protein Corona for Drug Delivery. ACS Appl. Nano Mater..

[41] Yeo E.L.L., Cheah J.U.J., Lim B.Y., Thong P.S.P., Soo K.C., Kah J.C.Y. (2017). Protein Corona around Gold Nanorods as a Drug Carrier for Multimodal Cancer Therapy. ACS Biomater. Sci. Eng..

[42] Albiter E., Alfaro S., Valenzuela M.A. (2015). Photosensitized oxidation of 9,10-dimethylanthracene with singlet oxygen by using a safranin O/silica composite under visible light. Photochem. Photobiol. Sci..

[43] Chen S., Zhao X., Chen J., Chen J., Kuznetsova L., Wong S.S., Ojima I. (2010). Mechanism-Based Tumor-Targeting Drug Delivery System. Validation of Efficient Vitamin Receptor-Mediated Endocytosis and Drug Release. Bioconjug. Chem..

[44] Choi B., Ryoo I., Kang H.C., Kwak M.-K. (2014). The sensitivity of cancer cells to pheophorbide a-based photodynamic therapy is enhanced by Nrf2 silencing. PLoS ONE.

[45] Tang P.M.-K., Liu X.-Z., Zhang D.-M., Fong W.-P., Fung K.-P. (2009). Pheophorbide a based photodynamic therapy induces apoptosis via mitochondrial-mediated pathway in human uterine carcinosarcoma. Cancer Biol. Ther..

