# Supplementary material for: Pheophorbide A and Paclitaxel Bioresponsive Nanoparticles as Double-Punch Platform for Cancer Therapy
Source: Pharmaceutics. 2021 Jul 23;13(8):1130. doi: 10.3390/pharmaceutics13081130 (PMC8399365; doi:10.3390/pharmaceutics13081130)
Supplement: Supplementary file 1 [file pharmaceutics-13-01130-s001.zip › pharmaceutics-1296890-supplementary.pdf]

pharmaceutics

Supporting Information: Pheophorbide A and Paclitaxel bioresponsive nanoparticles as double-punch platform for cancer therapy

Francesca Moret, Luca Menilli, Manuele Battan, Daniele Tedesco, Marta Columbaro, Andrea Guerrini, Greta Avancini, Claudia Ferroni and Greta Varchi

Table S1. Optimization studies on PheoA@PTX2S nanoparticles using different PheoA/PTX₂S ratio and different concentrations of PheoA in DMSO. 2.

$[PTX_2S]$ (mg/mL_{DMSO})	[PheoA] (mg/mL_{DMSO})	% PheoA (w_{PheoA}/w_{PTX2S})	Hydrodynamic Diameter (nm)	PDI
10	0.5	15	147 ± 1	0.17 ± 0.02
		20	157 ± 1	0.14 ± 0.01
		25	142 ± 6	0.15 ± 0.01
	1.5	30	61 ± 2	0.12 ± 0.03
		35	93 ± 3	0.11 ± 0.01
		40	78 ± 2	0.10 ± 0.02

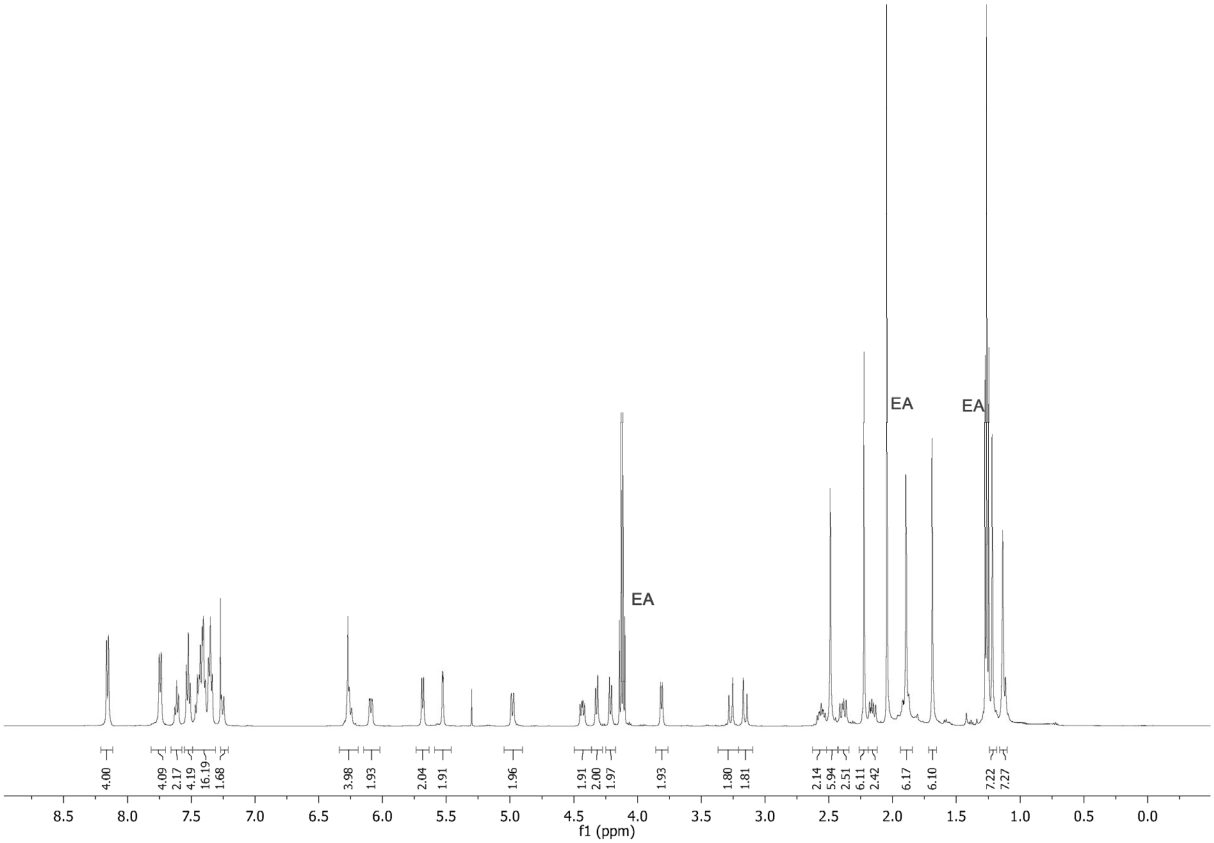

Figure S1. ^{1}H-NMR of PTX_2S in $CDCl_3$ recorded on 500 MHz Varian spectrometer. 2.

Figure S2. PheoA@PTX2S nanoparticles disassembly studies under different conditions: **a**) in the presence of 10 mM GSH at 37 °C for 24 h and **b**) in the presence of different H_2O_2 concentrations and plus light irradiation. 3.

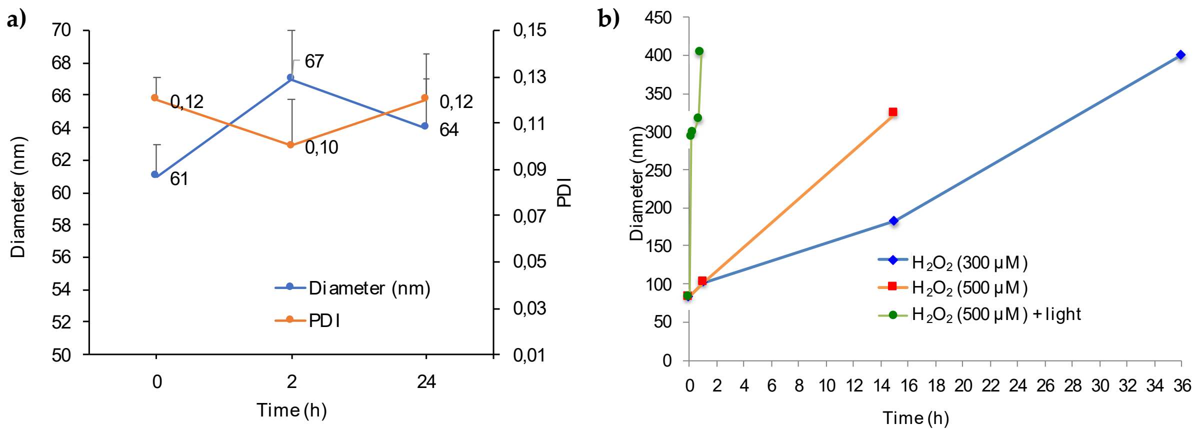

Figure S2. PheoA@PTX2S nanoparticles disassembly studies under different conditions: **a**) in the presence of 10 mM GSH at 37 °C for 24 h and **b**) in the presence of different H_2O_2 concentrations and plus light irradiation.

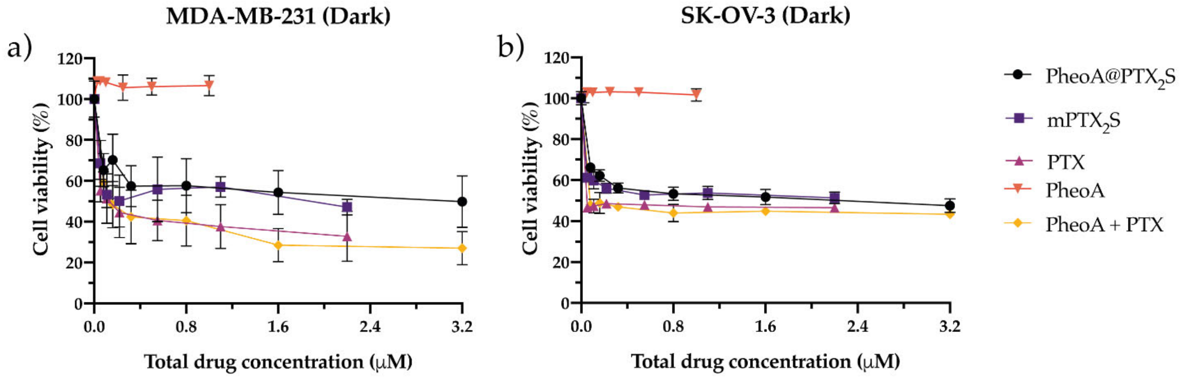

Figure S3. Dark cytotoxicity in vitro. Dose-response curves of MDA-MB-231 **a**) or SK-OV-3 **b**) cells incubated in the dark for 24 h with single drugs or their combination delivered free or in nanoparticles and released for additional 24 h in drug-free medium before cell viability measurement with MTS assay. Total drug concentration is referred to PTX + PheoA concentration. Data are expressed as mean percentage ± SD of at least three independent experiments, carried out in triplicate.

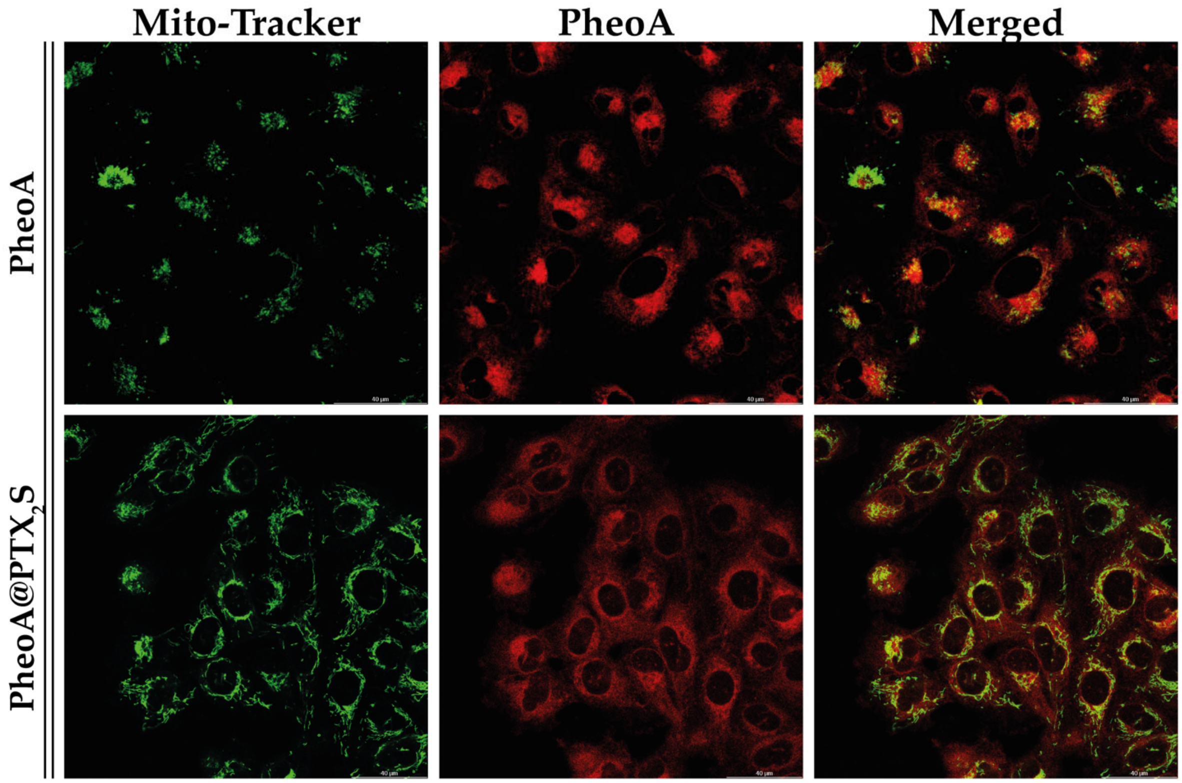

Figure S4. In vitro intracellular localization studies. Confocal microscopy images of MDA-MB-231 cells showing only slight co-localization between the red fluorescence of PheoA (delivered in the standard solvent or loaded in PheoA@PTX$_2$S) and the green fluorescence of MitoTracker used as specific probe for mitochondria. Scale bars: 40 μm.

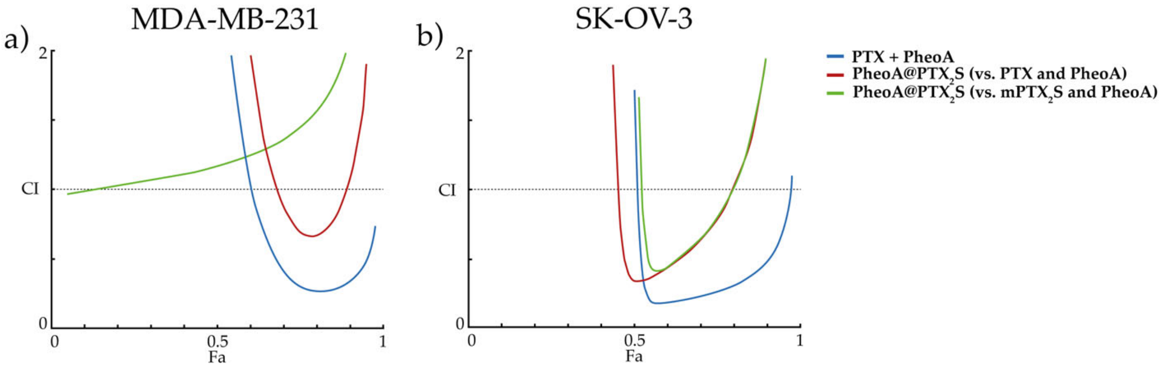

Figure S5. Combination index analysis. Plots of Combination Index vs. Fa (Fa-CI plot) relative to MDA-MB-231 **a**) and SK-OV-3 **b**) cells treated with the combination of PTX and PheoA. Data reported in Fig. 5 of the main text were analyzed with the Compusyn software and Fa-CI plots derived.
